# Association Between Health Literacy and One-Year Incidence of Pre-Frailty Among Older Adults Undergoing Frailty Health Checkups

**DOI:** 10.3390/geriatrics11030064

**Published:** 2026-05-21

**Authors:** Yoshiharu Yokokawa, Keisuke Nakamura, Tomohiro Sasaki, Shinobu Yokouchi

**Affiliations:** 1 School of Health Sciences, Faculty of Medicine, Shinshu University, Matsumoto 390-8621, Japan; keipons08@shinshu-u.ac.jp; 2 Health Promotion Division, Matsumoto City Hall, Matsumoto 390-8620, Japan; tomohiro_sasaki@city.matsumoto.lg.jp (T.S.); shinobu_yokouchi@city.matsumoto.lg.jp (S.Y.)

**Keywords:** frailty, pre-frailty, health literacy, predictors, inverse probability weighting

## Abstract

**Objective**: We aimed to identify predictors of the transition to pre-frailty among older adults undergoing frailty health checkups and to evaluate their relative importance. **Methods:** A longitudinal analysis was conducted using data from a frailty health checkup project involving 830 older adults participating in community exercise classes in Matsumoto City, Nagano Prefecture, Japan. Among them, 502 individuals classified as robust (healthy) at baseline were followed for 1 year. Predictors of transition to pre-frailty were examined using multivariate logistic regression analysis. Given the low follow-up rate (43.4%), a sensitivity analysis using inverse probability weighting (IPW) was performed. Model performance was internally validated using bootstrap resampling and calibration assessment. **Results:** Of the 218 participants who completed the follow-up period, 45 (20.6%) transitioned to pre-frailty. Increasing age was a significant risk factor (OR = 1.13, 95% CI: 1.06–1.20), whereas higher health literacy was a protective factor (OR = 0.51, 95% CI: 0.29–0.86). In the IPW sensitivity analysis, the association with age remained stable, while health literacy demonstrated borderline significance (*p* = 0.08). The model showed acceptable discrimination (area under the curve [AUC] = 0.737; 0.728 after optimism correction) and good calibration (Hosmer–Lemeshow test, *p* = 0.124). **Conclusions:** Age and health literacy were independent predictors of transition to pre-frailty. As a modifiable factor, health literacy may represent a promising target for interventions aimed at preventing the progression to pre-frailty.

## 1. Introduction

Japan is the most aged country in the world, with approximately 36 million people aged 65 years and older as of 1 October 2023, accounting for 29.1% of the total population [1]. In such a super-aged society, frailty has emerged as a significant public health challenge. Frailty is defined as a state of increased vulnerability to stressors due to age-related decline in physiological reserves [2] and is associated with increased risk of disability and mortality [3].

The physical frailty phenotype proposed by Fried et al. (Cardiovascular Health Study criteria) defines frailty as meeting three or more of five criteria: (1) weight loss, (2) exhaustion, (3) low physical activity, (4) slow walking speed, and (5) weak grip strength, while pre-frailty is defined as meeting one to two criteria [2]. The prevalence of frailty among Japanese older adults is estimated at 7–10%, and pre-frailty at 40–50% [4,5], indicating that a substantial proportion of older adults are at risk of progressing to frailty.

Frailty is strongly associated with adverse outcomes such as falls, hospitalization, institutionalization, and mortality [3,6,7,8]. According to a meta-analysis by Li et al., the non-robust group had a significantly higher risk of all-cause mortality than the robust group. Additionally, approximately half of community-dwelling older adults were classified as pre-frail, showing a 38% increase in all-cause mortality risk [7,8]. According to a meta-analysis by Vermeieren et al., older adults with frailty have approximately twice the mortality risk and approximately 1.5 times the hospitalization risk compared to non-frail older adults [6]. Furthermore, the increase in healthcare and long-term care costs attributable to frailty has become a serious social burden, and the importance of frailty prevention has been emphasized for extending healthy life expectancy and optimizing social security costs [9].

Many studies have been conducted on risk factors for frailty, and advanced age, female sex, sarcopenia, malnutrition, comorbid chronic diseases, depressive symptoms, cognitive decline, and social isolation have been identified [2,3,10]. In recent years, health literacy, defined as the ability to obtain, understand, and utilize health information, has attracted attention as a determinant of frailty. Low health literacy has been reported to be associated with increased mortality risk in older adults [11,12].

Pre-frailty, the precursor stage of frailty, is considered a reversible condition, and it is believed that appropriate interventions can enable a return to a robust state [13]. Predictors of transition to pre-frailty include aging, decreased physical activity, depressive symptoms, cognitive decline, and reduced social participation [14,15]. However, few studies have longitudinally examined the association between health literacy and transition to pre-frailty.

In Japan, guidelines for health services for the late-stage elderly were established in 2019, outlining national efforts toward frailty prevention [16]. Frailty has been positioned as “a reversible condition with appropriate intervention,” and efforts have been made to establish a comprehensive prevention system integrating medical care, long-term care, health services, and social participation. The aim is to create a system that comprehensively supports older adults at risk of frailty by combining health services under medical insurance, care prevention programs under long-term care insurance, and medical care provided by primary care physicians. As part of municipal programs, frailty screening is conducted with the aim of early identification of individuals at high risk of transitioning to pre-frailty and implementing efficient interventions. According to the Health Literacy Skills Framework [17], individuals with high health literacy excel in accessing, understanding, evaluating, and utilizing health information, and are considered to more effectively practice preventive behaviors such as proper nutrition, regular physical activity, and social participation—all of which are established protective factors against frailty [17]. Furthermore, Nutbeam’s model (2000) suggests that older adults with higher communicative and critical health literacy are more likely to actively utilize healthcare services and autonomously maintain health-promoting behaviors [18]. Despite this theoretical rationale, longitudinal evidence regarding the protective role of health literacy in preventing transition to pre-frailty among community-dwelling older adults undergoing community-based frailty screening remains limited. Since health literacy can be improved through educational interventions [19], clarifying this association is meaningful for positioning health literacy as a target for new preventive strategies.

The aim of this study was to identify predictors of pre-frailty and evaluate their relative importance among older adults undergoing frailty screening. The findings of this study are expected to contribute to the development of effective intervention strategies for optimizing frailty prevention and management programs in the target population.

## 2. Materials and Methods

### 2.1. Participants

The participants were 830 individuals enrolled in a government-administered frailty health checkup project initiated in 2022 in Matsumoto City, Nagano Prefecture, who participated in community exercise classes for older adults. The project conducted frailty assessments using questionnaires and evaluated physical function, including walking speed and grip strength, to identify individuals with frailty or at risk of frailty and to provide recommendations for appropriate interventions.

### 2.2. Study Design

A longitudinal study design was employed, and participants were followed for 1 year to assess changes in frailty status.

### 2.3. Measurement Items

The following variables were assessed, consistent with the measurement items reported in a previous study [20].

#### 2.3.1. Demographic Characteristics and Health-Related Variables

These included sex, age, subjective economic status, years of education, number of household members, number of comorbidities, grip strength, history of falls, history of health checkups, sleep duration, employment status, and total medical costs in 2022.

#### 2.3.2. Walking Speed and Grip Strength

Walking speed was calculated by measuring the time required to walk a 5 m straight path at a usual pace. Grip strength was measured in a standing position using a digital grip strength dynamometer (TKK-5401, SANKA Co. Ltd., Sanjyo, Japan).

#### 2.3.3. Frailty Classification

Frailty status was determined based on Category 3 of the revised Japanese version of the Cardiovascular Health Study criteria (revised J-CHS criteria) [5]. Participants meeting three or more of the five criteria were classified as frail, those meeting one to two criteria as pre-frail, and those meeting zero criteria as robust.

#### 2.3.4. Cognitive Function

Cognitive function was assessed using three items from the 25-item Kihon Checklist, a screening index designed to evaluate daily life functions [21]. This was used not for diagnostic purposes but to confirm the risk of cognitive decline within the limited time available for frailty screening. The assessment items were as follows: ➀ “Do others say that you are forgetful (e.g., asking the same thing repeatedly)?”, ➁ “Can you look up a phone number and make a call?”, and ➂ “Are there times when you do not know what month and day it is?”. Scores were calculated by summing the responses to each item.

#### 2.3.5. Health Literacy

Health literacy was measured using the Communicative and Critical Health Literacy Scale (5 items), which assesses the ability to obtain, understand, and utilize health-related information [22]. Each item was rated on a 5-point Likert scale from “do not agree at all” to “strongly agree,” and the mean of the total score (5–25 points) was used for analysis.

#### 2.3.6. Instrumental Activities of Daily Living (IADL)

IADL was assessed using five items from the Tokyo Metropolitan Institute of Gerontology Index of Competence (TMIG-IC) [23]. Each item was scored as 1 (yes) or 0 (no), and a total score (0–5 points) was calculated.

#### 2.3.7. Social Participation Ability

Social participation ability was assessed using the 4-item subscale on social participation from the JST Index of Competence [24]. Participation in community activities and engagement in social roles were evaluated, with each item scored as 1 point for a total of 0–4 points. The assessment items were: (1) participation in local festivals and events, (2) active involvement in neighborhood associations and community organizations, (3) assuming roles and responsibilities in community activities, and (4) participation in volunteer and service activities.

#### 2.3.8. Information Collection Ability

Information collection ability was assessed using another 4-item subscale on information collection from the JST Index of Competence [24]. The ability to collect and utilize information in daily life was measured, with each item scored as 1 (yes) or 0 (no) to calculate a score of 0–4 points. The assessment items were: interest in domestic and international news, ability to judge the reliability of health-related information, interest in cultural activities (art, movies, music, etc.), and viewing educational and cultural programs.

### 2.4. Statistical Analyses

A total of 502 participants classified as robust at baseline were included in the analysis. To compare baseline characteristics between those who completed the 1-year follow-up and those who dropped out, the Wilcoxon rank-sum test was used for continuous variables, and Pearson’s chi-square test or Fisher’s exact test was used for categorical variables. The magnitude of between-group differences was assessed using the standardized mean difference (SMD), with SMD < 0.1 indicating good balance and SMD ≥ 0.1 indicating meaningful imbalance [25].

Multivariate logistic regression analysis was performed to identify predictors of transition to pre-frailty. The dependent variable was transition to pre-frailty at 1 year (yes/no), and the independent variables were the three variables of age, health literacy, and social participation. These variables were selected because: (1) it was necessary to limit to three variables due to the EPV ≥ 10 constraint, (2) significant associations with pre-frailty transition had been demonstrated in our previous studies [20,26], and (3) each variable represents the three main components of frailty: physical (age-related changes through age), cognitive (health judgment ability through health literacy), and social (social participation) aspects [3]. Cognitive function (3 items from the Kihon Checklist), IADL, and fall history were considered candidate variables; however, they were excluded from the final model due to highly skewed distributions at baseline (e.g., 82.0% had full IADL scores). Given 45 outcome events, a model limited to three variables was adopted to ensure an events per variable (EPV) ratio of 10 or greater to reduce the risk of overfitting. Because both health literacy (CCHL) and information collection ability (JST subscale) are closely related constructs, Spearman’s rank correlation coefficient was calculated between the two variables (ρ = 0.29, *p* < 0.001). Although a correlation was observed between the two variables, in this study, CCHL was adopted as the explanatory variable in the final model because: (1) CCHL is a highly valid scale that directly assesses the functional concepts of communicative and critical health literacy [22], (2) Cronbach’s alpha coefficient was higher for CCHL (α = 0.85) than for information collection ability (α = 0.5), and (3) it is a scale that directly corresponds to the theoretical framework of this study (Squiers framework [17], Nutbeam model [18]). VIF was <1.5 for all variables, and multicollinearity was not a concern.

Because the follow-up rate was low (43.4%) and selection bias was a concern, sensitivity analysis using IPW was performed. Propensity scores for follow-up completion were estimated using logistic regression with age, sex, IADL, health literacy, social participation, and cognitive function as predictor variables. The inverse of the estimated propensity scores was used as weights. The propensity score model included all 502 participants (218 completers and 284 dropouts), and inclusion of six variables was considered statistically appropriate. To mitigate the influence of extreme weights, stabilized weights trimmed at the 1st to 99th percentiles were used.

Bootstrap methods were used to assess internal validity. The stability of odds ratio (OR) estimates was assessed using 1000 bootstrap iterations. Model discrimination was assessed using the C-statistic (AUC), with optimism correction performed using 200 bootstrap iterations. Calibration was assessed using the Hosmer–Lemeshow test, calibration plots by deciles of predicted risk, and mean absolute error (MAE) [27].

All statistical analyses were performed using R version 4.5.3 (R Foundation for Statistical Computing, Vienna, Austria), with a two-sided significance level of 5%.

### 2.5. Ethical Considerations

This study was conducted with approval from the Ethics Committee of Matsumoto City Hospital (Approval No.: 03-5). As this study was conducted as part of the city’s preventive health checkup program targeting frailty risk, consent was obtained through an opt-out method in accordance with institutional ethical guidelines. Participants were informed in advance about the opportunity to decline participation through written materials distributed to eligible individuals and explanatory materials provided at the frailty checkup. Participants were informed that they were free to withdraw from the study.

## 3. Results

### 3.1. Participant Characteristics

**Figure 1:**
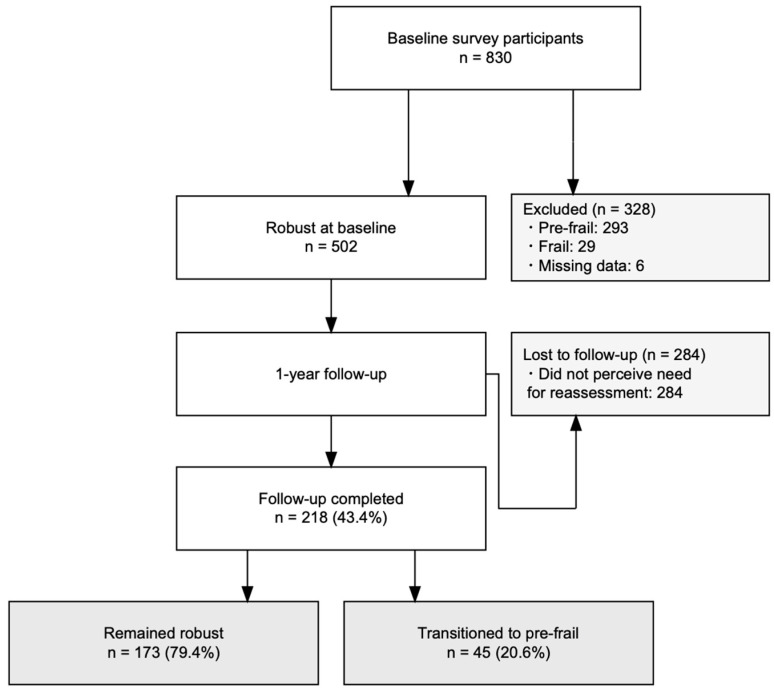
Flow diagram for participant selection and follow-up.

Of the 830 individuals assessed at baseline, 502 were classified as robust and included in the analysis (Figure 1). The 328 excluded individuals consisted of 293 with pre-frailty, 29 with frailty, and 6 with missing data. At the 1-year follow-up, 218 participants (43.4%) completed the assessment, and 284 (56.6%) dropped out. The main reasons for dropout were personal circumstances and feeling that participation in the first-year reassessment was unnecessary.

Table 1 shows the comparison of baseline characteristics between follow-up completers and dropouts. No significant differences were observed between the two groups for age (76 years vs. 77 years, *p* = 0.689), number of comorbidities (*p* = 0.219), years of education (*p* = 0.28), subjective economic status (*p* = 0.378), and history of health checkups (*p* = 0.136). On the other hand, IADL scores (mean ± SD 4.76 ± 0.78 vs. 4.93 ± 0.26, *p* = 0.003, SMD = 0.285) and social participation (mean ± SD 2.39 ± 1.43 vs. 2.73 ± 1.78, *p* = 0.006, SMD = 0.248) were significantly higher in follow-up completers. Although the medians were identical, assessment by SMD indicated a meaningful imbalance, suggesting the presence of potential selection bias.

### 3.2. Incidence of Transition to Pre-Frailty

Of the 218 participants who completed the 1-year follow-up, 173 (79.4%) maintained robust status, and 45 (20.6%) transitioned to pre-frailty (Figure 1, see Table S1). No participants progressed to frailty during the follow-up period.

### 3.3. Predictors of Transition to Pre-Frailty

Multivariate logistic regression analysis revealed that age (OR = 1.13, 95% CI: 1.06–1.20, *p* < 0.001) and health literacy (OR = 0.51, 95% CI: 0.29–0.86, *p* = 0.01) were significantly associated with transition to pre-frailty (Table 2). Age was a risk factor, with a 13% increase in the odds of transitioning to pre-frailty for each one-year increase in age. In contrast, health literacy was a protective factor, with a 49% decrease in transition odds for each one-point increase. Social participation did not show a significant association with transition to pre-frailty (OR = 1.12, 95% CI: 0.85–1.50, *p* = 0.44).

### 3.4. Sensitivity Analysis for Dropout Bias

In the sensitivity analysis using IPW, the effect of age was also maintained stably (OR = 1.13, 95% CI: 1.06–1.20, *p* < 0.001) (Table 2). Health literacy showed borderline significance after weighting (OR = 0.55, 95% CI: 0.29–1.06, *p* = 0.08), but the direction and magnitude of the effect remained largely unchanged. Social participation did not show a significant association even after IPW adjustment (OR = 1.14, 95% CI: 0.86–1.53, *p* = 0.36). The mean stabilized IPW weight was 0.983 (theoretical value: 1.0), with a range of 0.713–2.129 before trimming. Three cases (1.4%) exceeded the 99th percentile threshold (1.903), and trimming was performed. The mean weight after trimming was 0.981 (range: 0.732–1.903), and the impact of trimming on the overall weight distribution was minimal. The small proportion of extreme weights indicates that the correction for attrition bias using IPW was performed stably.

### 3.5. Model Validation

The stability of OR estimates was confirmed through 1000 bootstrap iterations (Figure S1). The bootstrap 95% confidence interval for age was 1.06–1.20, and the distribution did not include OR = 1. The majority of the bootstrap distribution for health literacy was below OR = 1, supporting the robustness of the protective effect.

Solid vertical lines indicate point estimates from the original model, and dashed vertical lines indicate an OR of 1 (null effect).

**Figure 2:**
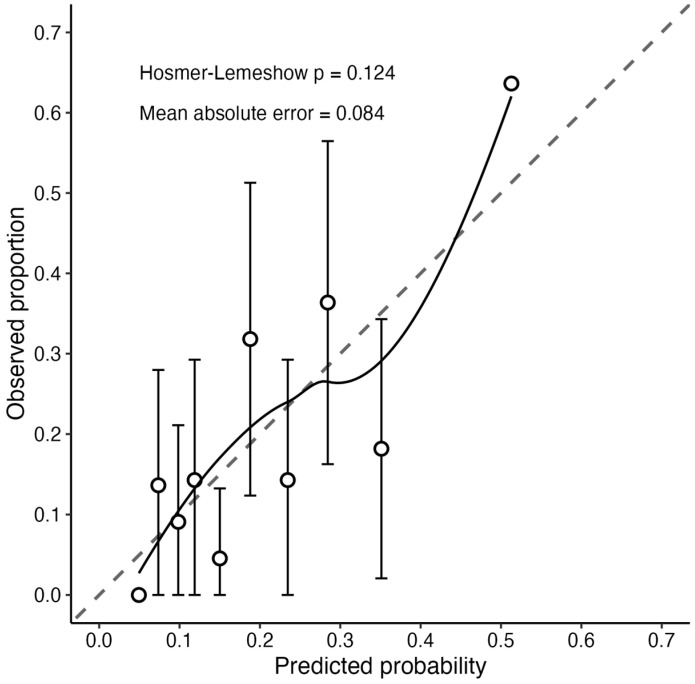
Calibration plot for the prediction model of transition to pre-frailty. The dashed diagonal line represents perfect calibration. Each point indicates the observed proportion within deciles of predicted probability, and error bars represent 95% confidence intervals.

Model discrimination was within an acceptable range, with a C-statistic of 0.737 (95% CI: 0.657–0.818) and 0.728 after optimism correction (Table 3). Calibration demonstrated good agreement between predicted and observed values, as indicated by the Hosmer–Lemeshow test (*p* = 0.124) and MAE (0.084) (Figure 2). The Brier score and Nagelkerke R^2^ were 0.144 and 0.173, respectively.

## 4. Discussion

### 4.1. Summary of Results

In this study, we followed 502 community-dwelling robust older adults for one year to examine predictors of transition to pre-frailty. Of the 218 participants who completed the follow-up, 45 (20.6%) transitioned to pre-frailty. Multivariate logistic regression analysis identified aging as a risk factor (OR = 1.13) and higher health literacy as a protective factor (OR = 0.51). To address the high attrition rate (56.6%), sensitivity analysis using IPW was conducted, and the internal validity of the model was confirmed through bootstrap methods and calibration assessment.

### 4.2. Predictors of Transition to Pre-Frailty

Our finding that aging is associated with the risk of transition to pre-frailty is consistent with previous studies. Fried et al. reported that the prevalence of frailty increases sharply after age 75 [2], and our longitudinal results support this finding. Age-related decline in muscle strength, decline in physiological reserve, and decline in homeostatic maintenance function are considered the main mechanisms underlying progression to pre-frailty [3].

The finding that health literacy functions as a protective factor against transition to pre-frailty is an important contribution of this study. Health literacy is defined as the ability to obtain, understand, and utilize health-related information [28]. Older adults with higher health literacy are more likely to understand and implement appropriate guidance on exercise and nutrition, which are essential for frailty prevention. Several mediating pathways are hypothesized as specific mechanisms by which health literacy inhibits transition to pre-frailty. First, older adults with higher health literacy may be able to understand and implement information regarding protein intake and nutritional balance necessary for frailty prevention. Second, appropriately determining the recommended physical activity levels and types of exercise may lead to continued participation in community exercise classes. Third, they may actively utilize medical and health services, leading to early detection of health problems and healthcare-seeking behavior [18]. Although this study design did not allow direct examination of these mediating variables, future studies should quantitatively evaluate these pathways using mediation analysis. It should be noted that health literacy may also be a proxy indicator for cognitive function or socioeconomic status, and replication studies including these as adjustment variables are warranted. Since health literacy influenced the development of pre-frailty at one year, it may function as a factor that prevents pre-frailty in healthy older adults. Consistent with our results, Fan et al. reported that low health literacy is associated with increased mortality risk in older adults [11]. Similarly, Choi et al. reported in a longitudinal cohort study of Korean older adults that low health literacy increased the risk of developing pre-frailty by approximately 1.4 times [29]. Furthermore, a retrospective cohort study using the same data from the Matsumoto City Frailty Screening Project showed that higher health literacy was associated with a reduced risk of frailty progression at one year, with IADL as a significant mediating factor [26].

Social participation showed between-group differences in univariate analysis (SMD = 0.213), but it was not a significant predictor in the multivariate model. This is because the independent effect of social participation was attenuated after adjusting for age and health literacy. The study participants may have delayed their transition to pre-frailty through having higher literacy that enabled them to understand the meaning of health behaviors, rather than through participation in public activities to maintain socially productive activities. Since multicollinearity was low, this is considered to be due to the independent effects of the variables. Research on social frailty has shown that continuing hobby activities involving others, such as exercise, prevents progression to pre-frailty and frailty [30]. Although social participation and health literacy are conceptually different, previous studies have reported that older adults with higher levels of social participation tend to have higher health literacy [31]. Participation in social activities may increase opportunities for accessing and utilizing health-related information, leading to improved health literacy; however, the causal relationships between these factors require further examination in future longitudinal studies.

### 4.3. Sensitivity Analysis

In this study, the follow-up rate was low (43.4%), and differences were observed between those who completed follow-up and those who dropped out in IADL (SMD = 0.285) and social participation (SMD = 0.248). When such selection bias exists, the results of standard regression analysis may be distorted [32]. To address this issue, sensitivity analysis using IPW was conducted.

The effect of age remained stable after applying IPW (OR = 1.13, *p* < 0.001), suggesting that it was not affected by attrition bias. In contrast, the association with health literacy changed from *p* = 0.01 to *p* = 0.08, showing borderline significance. The variation in *p*-values suggests that while the direction of the effect is robust, the strength of the protective effect of health literacy may have been slightly overestimated in the standard model due to higher-functioning individuals remaining in the study.

Selection bias due to attrition threatens the internal validity of cohort studies, and IPW is recommended as a standard method to correct for this bias [33]. In this study, the fact that the change in OR before and after applying IPW was less than 10% supports the robustness of the results [33].

### 4.4. Validity Verification

The internal validity of the model was evaluated using multiple indices. The discrimination assessed by the C-statistic (AUC) was 0.737, which remained stable at 0.728 after optimism correction, falling within the acceptable range (0.7–0.8) [27]. This indicates that the model can appropriately discriminate between participants with high and low risk of transition to pre-frailty.

Calibration was assessed as good by the Hosmer–Lemeshow test (*p* = 0.124). In the calibration plot, the predicted probabilities and observed proportions showed good agreement along the 45-degree line, with an MAE of 0.084. This indicates that the predicted probabilities of the model accurately reflect the actual incidence rates [34].

The stability of the OR estimates for age and health literacy was confirmed through 1000 bootstrap iterations. The bootstrap 95% confidence intervals approximated the Wald-based confidence intervals, suggesting that estimation instability due to the relatively small sample size was minimal.

The Brier score and Nagelkerke R^2^ were 0.144 and 0.173, respectively, indicating limited explanatory power at the individual level. However, these values are within the range commonly observed in studies targeting multifactorial health outcomes such as frailty transition. The primary purpose of this model is to identify risk factors at the population level and evaluate their relative importance, rather than to provide precise individual predictions. Based on the acceptable discrimination (AUC = 0.737) and good calibration (Hosmer–Lemeshow test, *p* = 0.124), clinical utility is considered appropriate. On the other hand, this implies that caution is needed when interpreting the clinical utility of the model as an individual-level prediction tool. Steyerberg et al. emphasized the importance of reporting both discrimination and calibration in the development of prediction models, and this study followed this recommendation [35]. A recent systematic review of frailty risk prediction models for community-dwelling and hospitalized older adults (22 models) reported that the AUC for internal validation ranged from 0.707 to 0.920 [36], and the discrimination of our model is within the acceptable range reported in the literature. Additionally, age was the most frequently identified predictor in 77.3% of all models, which is consistent with the findings of this study.

### 4.5. Clinical Implications

The findings of this study provide the following three implications for the design of preventive interventions in community frailty screening. First, since health literacy is an independent protective factor against transition to pre-frailty, implementing health education programs that support older adults in understanding, evaluating, and utilizing information about signs of frailty, prevention methods, and appropriate nutrition and exercise at frailty screening sites is considered effective. Second, based on Nutbeam’s communicative and critical health literacy model [18], it is recommended to hold participatory learning classes that include interactive communication and development of decision-making skills, rather than simply providing information. Third, since health literacy is a modifiable factor unlike age, it is positioned as an important intervention target for primary prevention in the community-based integrated care system. Specific interventions may include (1) individual health guidance using the teach-back method to confirm understanding of health information; (2) small-group learning classes with multidisciplinary collaboration among community public health nurses, dietitians, and physical therapists; (3) health information literacy improvement programs utilizing ICT; and (4) regular health literacy assessment and feedback through collaboration between community exercise classes and health screenings.

### 4.6. Study Limitations

This study has several limitations.

First, the follow-up rate was low (43.4%). The main reasons for dropout were personal circumstances and feeling that participation in the first-year reassessment was unnecessary, and dropout due to deterioration of health status is considered to be rare. The dropout rate of 56.6% is relatively high, and dropout bias may have occurred. IPW was applied to adjust for measured confounders associated with dropout, but this method cannot completely adjust for unmeasured reasons for dropout, such as rapid health deterioration that could not be captured by baseline IADL assessment. This limitation must be considered when interpreting the findings of this study.

Second, the number of events was small (*n* = 45), limiting statistical power. A simplified model with three variables was adopted to meet the EPV requirement, but the independent effects of other potential predictors such as cognitive function, IADL, and fall history may not have been sufficiently examined. Replication studies in larger cohorts are needed.

Third, this study was conducted in a single region, and external validity has not been verified. The participants were older adults participating in community exercise classes in Matsumoto City, Nagano Prefecture, and caution is needed when generalizing to older adults not participating in exercise classes or populations in other regions. Verification of external validity is a future challenge in multicenter collaborative studies.

Fourth, the follow-up period is short at 1 year. A longer follow-up is needed to evaluate more serious outcomes such as progression to frailty and death.

Fifth, the measurement of health literacy was limited. This study used a self-administered questionnaire and did not verify the relationship with actual health information utilization behavior. The causal relationship between health literacy and social participation could not be clarified with this study design. If standardized scales such as MMSE or MoCA had been used, different results regarding the relationship between cognitive function and health literacy might have been obtained. Additionally, this study included only participants classified as robust at baseline and longitudinally evaluated the occurrence of pre-frailty after 1 year, thereby ensuring temporal sequence. However, even in the absence of baseline manifestation, subclinical physiological changes (e.g., mild inflammation, early cognitive function changes) may have influenced both health literacy and transition to pre-frailty. In future studies, it is necessary to more precisely examine the mechanisms of the protective effect of health literacy through repeated measurements, including biological markers and mediation analysis, among other methods.

Sixth is selection bias. The participants in this study were older adults who voluntarily participated in community exercise classes in Matsumoto City and may have higher health awareness and be more physically active compared to the general community-dwelling older adult population. The possibility that the risk of transition to pre-frailty in this sample is underestimated due to this “healthy volunteer effect” cannot be ruled out. Direct comparison data with the general older adult population in Matsumoto City were not available in this study, but compared with national reference data [37] (Murayama et al., 2020; *n* = 2206), the proportion of robust individuals in the baseline data (60.9%) was higher than the national estimate (50.5%), and the proportion of frailty (3.5%) was lower than the national estimate (8.7%). Therefore, caution is required when extrapolating the results of this study to the general community-dwelling older adult population.

### 4.7. Study Strengths and Future Research

This study has several methodological strengths. First, by limiting the independent variables to 3 for the number of events (*n* = 45), the Events Per Variable ratio (EPV) was 15, meeting the recommended threshold for logistic regression stability (EPV ≥ 10). Second, in response to the high dropout rate of 56.6%, sensitivity analysis using IPW and internal validity verification through 1000 bootstrap iterations was conducted, confirming the robustness of the results. Third, this is one of the few studies to longitudinally examine health literacy as a predictor of transition to pre-frailty, providing a new perspective to frailty prediction research that has previously focused on physical function and social participation. Future research directions include (1) verification of external validity through multicenter collaborative studies in multiple regions, (2) evaluation of the impact on frailty onset and hard outcomes (long-term care certification, death) through long-term follow-up of 2 years or more, (3) concurrent use of cognitive function assessment using MMSE and other measures, and (4) verification of effectiveness through randomized controlled trials of intervention studies aimed at improving health literacy.

## 5. Conclusions

This study demonstrated that in community-dwelling older adults, aging is a risk factor for transition to pre-frailty, while high health literacy is a protective factor. While aging is a non-modifiable factor, health literacy is modifiable and may serve as a promising target for the prevention of transition to pre-frailty. Programs aimed at improving the ability of older adults to understand and utilize health information should be promoted as an effective strategy for preventing the onset of pre-frailty.

## Figures and Tables

**Table 1:** Baseline characteristics of robust participants by follow-up status (*n* = 502).

Variable	Not Completed (*n* = 284)	Completed Follow-Up (*n* = 218)	*p*	SMD
Age	77 (72–82)	76 (72–81)	0.689	0.019
Sex (female)	224 (78.9)	182 (83.4)	0.194	0.129
Number of comorbidities (*n* = 0)	182 (64.1)	151 (69.3)	0.219	0.224
Years of education			0.28	0.207
NA	9 (3.2)	4 (1.8)		
9 years	37 (13.0)	16 (7.3)		
12 years	161 (56.7)	130 (59.6)		
15 years	66 (23.2)	56 (25.7)		
Others	11 (3.9)	12 (5.5)		
Number of cohabitants			0.053	0.328
NA	7 (2.5)	0 (0.0)		
Couple	82 (28.9)	76 (34.9)		
Alone	58 (20.4)	59 (27.1)		
Couple and child	55 (19.4)	23 (10.6)		
Three-generation household	34 (11.9)	20 (9.2)		
Child	26 (9.2)	22 (10.1)		
Others	22 (7.7)	18 (8.3)		
Subjective economic status			0.378	0.208
NA	7 (2.5)	2 (0.9)		
Can afford daily living expenses	24 (8.5)	14 (6.4)		
Can afford to live a little	31 (10.9)	36 (16.5)		
Neither	203 (71.5)	151 (69.3)		
Can hardly afford to live	13 (4.5)	7 (3.2)		
Cannot afford daily living expenses	6 (2.1)	8 (3.7)		
Employment status (yes)	41 (14.4)	36 (16.5)	0.666	0.05
Health checkups (yes)	214 (75.4)	180 (82.6)	0.136	0.146
History of falls (yes)	43 (15.1)	33 (15.1)	1.000	0.008
Sleep duration	7 (6–7.5)	7 (6–8)	0.366	0.052
Grip strength (kg)	24.3 (21.0–28.4)	23.7 (20.7–27.9)	0.703	0.046
Walk speed (m/s)	1.28 (1.16–1.43)	1.31 (1.18–1.43)	0.212	0.094
Cognitive decline	1 (1–2)	1 (1–2)	0.696	0.048
IADL(median)	5 (5–5)	5 (5–5)	0.025	0.273
IADL(means)	4.76 ± 0.78	4.93 ± 0.26	0.003	0.285
Proportion with full IADL score (5 points)	220 (77.5%)	202 (92.7%)	<0.001	0.443
Health Literacy	4 (3.4–4.2)	4 (3.6–4.2)	0.209	0.155
Information collection	4 (3–4)	4 (3–4)	0.431	0.073
Social participation	3 (1–4)	3 (2–4)	0.021	0.213
Medical costs in 2022	274,055 (112,392–366,335)	212,390 (125,615–365,075)	0.83	0.095

NA: No Answer. Values are presented as median (IQR) or *n* (%).

**Table 2:** Multivariable logistic regression analysis of pre-frailty transition.

	Unweighted Model		IPW-Weighted Model	
Variable	OR (95% CI)	*p*	OR (95% CI)	*p*
Age (per 1-year increase)	1.13 (1.06–1.20)	<0.001	1.13 (1.06–1.20)	<0.001
Health literacy (per 1-point increase)	0.51 (0.29–0.86)	0.01	0.55 (0.29–1.06)	0.08
Social participation (per 1-point increase)	1.12 (0.85–1.50)	0.44	1.14 (0.86–1.53)	0.36

OR: odds ratio; CI: confidence interval; IPW: inverse probability weighting; IPW was used to adjust for potential selection bias due to loss to follow-up (56.6%).

**Table 3:** Model performance metrics.

Category	Metric	Value
Discrimination	C-statistic (AUC)	0.737
	95% CI	0.657–0.818
	Optimism	0.01
	Optimism-corrected AUC	0.728
Calibration	Hosmer–Lemeshow chi-square (df = 8)	12.65
	*p* value	0.124
	Mean absolute error	0.084
Overall	Brier score	0.1442
	Nagelkerke R^2^	0.173

AUC: area under the receiver operating characteristic curve; Optimism was estimated using 200 bootstrap samples.

## Data Availability

The data presented in this study are available on request from the corresponding author due to privacy and ethical restrictions.

## Supplementary Materials

The following supporting information can be downloaded at: https://www.mdpi.com/article/10.3390/geriatrics11030064/s1, Figure S1: Bootstrap distributions of odds ratios (1000 replicates); Table S1: Follow-up data.

## Abbreviations

The following abbreviations are used in this manuscript:

SMD	Standardized Mean Difference
IPW	Inverse Probability Weighting
AUC	Area Under the Curve
