# Supplementary material for: Association Between Health Literacy and One-Year Incidence of Pre-Frailty Among Older Adults Undergoing Frailty Health Checkups
Source: Geriatrics (Basel). 2026 May 21;11(3):64. doi: 10.3390/geriatrics11030064 (PMC13214775; doi:10.3390/geriatrics11030064)
Supplement: Supplementary file 1 [file geriatrics-11-00064-s001.zip › geriatrics-4224962-supplementary.pdf]

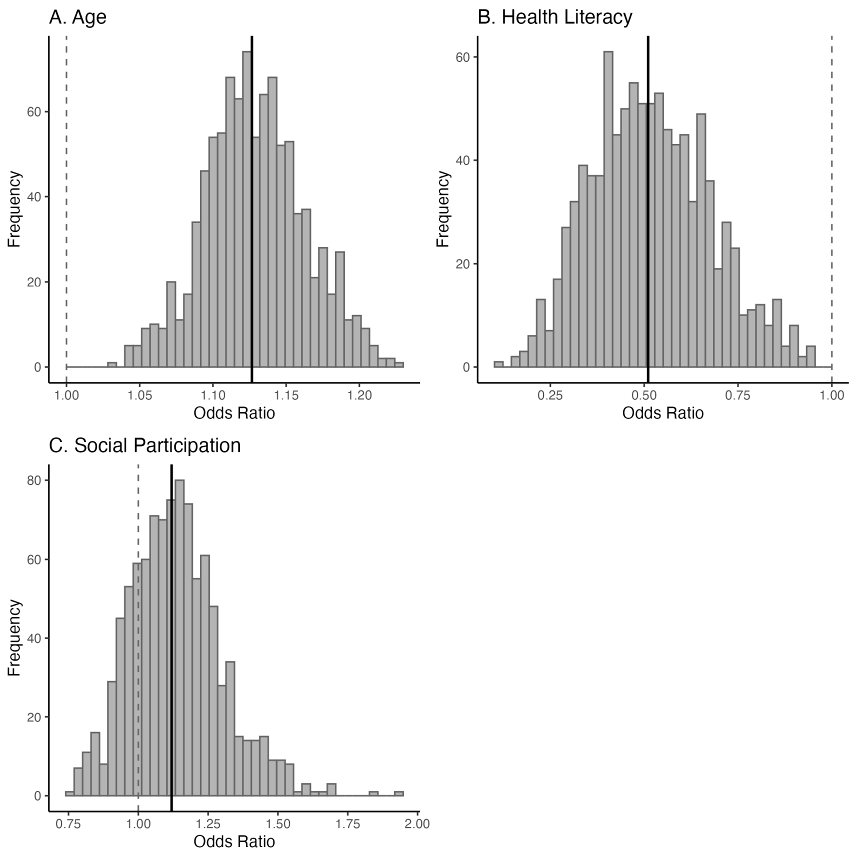

Figure S1. Bootstrap distributions of odds ratios (1,000 replicates)

Solid vertical lines indicate point estimates from the original model, and dashed vertical lines indicate an OR of 1 (null effect).

Table S1: Follow-up data.

Variable	Robust (n = 173)	Pre-frail (n = 45)	p	SMD
Age	75 (71–81)	81 (76–84)	<0.001	0.745
Sex (female)	144 (83.2)	38 (84.4)	1.000	0.033
Number of comorbidities (n = 0)	122 (70.5)	29 (64.4)	0.72	0.213
Years of education			0.318	0.371
NA	4 (2.3)	0 (0.0)		
9 years	10 (5.8)	6 (13.3)		
12 years	102 (58.9)	28 (62.3)		
15 years	47 (27.2)	9 (20.0)		
Others	10 (5.8)	2 (4.4)		
Number of cohabitants			0.033	0.615
Couple	64 (37.0)	12 (26.7)		
Alone	39 (22.5)	20 (44.4)		
Couple and child	19 (11.0)	4 (8.9)		
Three-generation household	19 (11.0)	1 (2.2)		
Child	16 (9.2)	6 (13.3)		
Others	16 (9.2)	2 (4.4)		

Subjective economic status			0.101	0.484
NA	2 (1.2)	0 (0.0)		
Can afford daily living expenses	12 (6.9)	2 (4.4)		
Can afford to live a little	32 (18.5)	4 (8.9)		
Neither	119 (68.8)	32 (71.1)		
Can hardly afford to live	4 (2.3)	3 (6.7)		
Cannot afford daily living expenses	4 (2.3)	4 (8.9)		
Employment status (yes)	33 (19.1)	3 (6.7)	0.076	0.377
Health checkups (yes)	144 (83.2)	36 (80.0)	0.772	0.084
History of falls (yes)	21 (12.1)	12 (26.7)	0.029	0.374
Sleep duration	7 (6–8)	7 (6–8)	0.795	0.072
Grip strength (kg)	24.3 (21.5–28.6)	20.7 (19.4–25.2)	<0.001	0.548
Walk speed (m/s)	1.3 (1.2–1.5)	1.2 (1.1–1.3)	<0.001	0.876
Cognitive decline	1 (1–2)	1 (1–2)	0.82	0.035
IADL	5 (5–5)	5 (5–5)	0.846	0.033
Health Literacy	4 (3.8–4.2)	3.6 (3.2–4)	<0.001	0.591
Information collection	4 (3–4)	4 (3–4)	0.259	0.188
Social participation	3 (2–4)	3 (2–4)	0.244	0.153
Medical costs in 2022	183100 (64130–334420)	210540 (109450–366000)	0.25	0.089

NA: No Answer. Values are presented as median (IQR) or n (%).
